# Complex Evaluation of Nanocomposite-Based Hydroxyapatite for Biomedical Applications

**DOI:** 10.3390/biomimetics8070528

**Published:** 2023-11-06

**Authors:** Daniela Predoi, Simona Liliana Iconaru, Steluta Carmen Ciobanu, Nicolas Buton, Mihai Valentin Predoi

**Affiliations:** 1 National Institute of Materials Physics, Atomistilor Street, No. 405A, MG 07, 077125 Magurele, Romania; ciobanucs@gmail.com; 2 HORIBA Jobin Yvon S.A.S., 6-18, Rue du Canal, 91165 Longjumeau, France; nicolas.buton@horiba.com; 3 Department of Mechanics, University Politehnica of Bucharest, BN 002, 313 Splaiul Independentei, Sector 6, 060042 Bucharest, Romania; predoi@gmail.com

**Keywords:** magnesium doped hydroxyapatite, chitosan, stable suspensions, antimicrobial activity, cytotoxicity

## Abstract

A magnesium-doped hydroxyapatite in chitosan matrix (MgHApC) sample was developed as a potential platform for numerous applications in the pharmaceutical, medical, and food industries. Magnesium-doped hydroxyapatite suspensions in the chitosan matrix were obtained by the coprecipitation technique. The surface shape and morphological features were determined by scanning electron microscopy (SEM). The hydrodynamic diameter of the suspended particles was determined by Dynamic light scattering (DLS) measurements. The stability of MgHApC suspensions was evaluated by ultrasonic measurements. The hydrodynamic diameter of the MgHApC particles in suspension was 29.5 nm. The diameter of MgHApC particles calculated from SEM was 12.5 ± 2 nm. Following the SEM observations, it was seen that the MgHApC particles have a spherical shape. The Fourier-transform infrared spectroscopy (FTIR) studies conducted on MgHApC proved the presence of chitosan and hydroxyapatite in the studied specimens. *In vitro* antimicrobial assays were performed on *Escherichia coli* ATCC 25922, *Staphylococcus aureus* ATCC 25923, *Pseudomonas aeruginosa* ATCC 27853, and *Candida albicans* ATCC 10231 microbial strains. The antimicrobial experiments showed that MgHApC exhibited very good antimicrobial properties against all the tested microorganisms. More than that, the results of the *in vitro* studies revealed that the antimicrobial properties of the samples depend on the incubation time. The evaluation of the sample’s cytotoxicity was performed using the human colon cancer (HCT-8) cell line. Our results suggested the great potential of MgHApC to be used in future applications in the field of biomedical applications (e.g., dentistry, orthopedics, etc.).

## 1. Introduction

In the last few years, tremendous progress has been made in the area of biomaterials, leading to the development of new and improved multifunctional materials with excellent biological and physico-chemical properties. Even though significant progress has been made regarding the design and secure use of materials with specific properties [1,2,3,4], there are still significant limitations in the current biomaterials-based strategies used in different fields such as medicine, dentistry, the food industry, orthopedic implants, tissue engineering, prosthetics, coatings, etc. [5,6,7,8,9,10,11]. During the years, research was focused on the development of novel biomaterials whose predominant function was to restore, substitute for, or improve the function of various organs or tissues in fields such as orthopedic surgery [4], plastic surgery [12], cardiac surgery [13], and stomatology [14]. One of the most promising candidates, often used in the fabrication of novel biocomposites, is hydroxyapatite (HAp). Hydroxyapatite, with the chemical composition Ca_10_(PO_4_)_6_(OH)_2_, is probably the main inorganic component found in human and animal bone and tooth tissues and is well known for its outstanding biological characteristics, such as bioactivity, biocompatibility, osteoconductivity, and nontoxicity [15,16,17,18]. Under these circumstances, composite materials based on hydroxyapatite-based materials have attracted research attention because of their biological features, which allow them to be applied for therapeutic purposes in implantology and dentistry. For example, the involvement of the biomimicry approach in the synthesis of new materials based on composite-hydroxyapatite can allow the obtaining of materials with an increased affinity for biological tissues, which is a very important property, especially for applications in the field of regenerative medicine [19,20]. According to previous studies, approaches such as biomineralization/bio-regeneration/ biomimicry may revolutionize the biomedical field (e.g., dentistry, orthopedics, etc.) and finally lead to significant improvements in human health [19,20].

As shown in previous studies, the use of dopants could lead to the improvement of both the physico-chemical and biological properties of hydroxyapatite nanoparticles. By improving these properties, hydroxyapatite could become the ideal candidate that could be used in obtaining new biocomposites used successfully in different biomedical applications [21,22,23,24]. Among the ions proposed as dopants for HAp, magnesium (Mg^2+^) is a chemical element with exceptional biological properties. A cation with a high abundance in the human body, magnesium, is involved in biological processes such as protein synthesis and glycolysis; it is involved in the activation of enzyme systems; and it also has an essential role in regulating the crystallization of the biological calcium phosphates [24,25,26]. Nonetheless, it has been reported that even though synthetic materials have outstanding biological properties, natural biomaterials derived either from plants or animals possess advantages over synthetic materials in that they have abundant availability and special biological activities [27,28]. A known natural biomaterial involved in various biomedical applications is chitin, which can be derived from both plants and animals and is one of the most abundant natural polymers found on earth [1,28,29]. Chitin is uniquely different from other polysaccharides due to the fact that it contains approximately 6% nitrogen in addition to oxygen, carbon, and hydrogen [30,31]. Despite its exquisite properties, because of its hydrophobicity [31,32], chitin cannot be used. Therefore, researchers are exploring chitosan, a product obtained by chitin deacetylation, which has many biological benefits such as biocompatibility, bioactivity, non-toxicity, biodegradability, and antimicrobial activity against a wide range of microorganisms [32,33,34].

Recently, a lot of attention has been focused on the area of dentistry and orthopedics because both the staff and the patients are exposed to an increased risk of infection by a wide range of microorganisms due to surgical procedures [34,35]. Therefore, in the past decade, great efforts have been made to develop new materials that have the ability to prevent and treat infectious diseases and also help to restore the original functions of affected tissues [34,35,36,37]. In the study conducted by Zhao et al. [34], it was shown that the presence of magnesium on a surface coated with Mg/HAp enhanced the osteogenic differentiation of pre-osteoblasts and the early osseointegration of bone [34]. Previously, the noncytotoxic effect of hydroxyapatite composite materials was highlighted using human colon cancer (HCT-8) and primary osteoblast culture (hFOB 1.19) cell lines [23]. It has been reported that the incorporation of Mg^2+^ into pulp-capping materials could be considered a new approach for developing materials that could enhance the regeneration of dental tissues [38]. And last but not least, it was observed that the presence of magnesium in compounds based on hydroxyapatite improves the *in vitro* antimicrobial activity of HAp [39].

Consequently, the main objective of this work was represented by the synthesis and complex evaluation from a physico-chemical and biological point of view of magnesium-doped hydroxyapatite in a chitosan matrix (Ca_10−x_Mg_x_(PO_4_)_6_(OH)_2_; x_Mg_ = 0.05; MgHApC). The results of our complex studies suggest that MgHApC possesses improved physico-chemical and biological features and therefore has a great potential for use in the development of novel biomedical applications (e.g., topical antiseptics, dentistry, orthopedics, etc.).

## 2. Materials and Methods

### 2.1. Materials

For the synthesis of magnesium-doped hydroxyapatite in chitosan matrix (MgHApC, Ca_10−x_Mg_x_(PO_4_)_6_(OH)_2_; x_Mg_ = 0.05), the sol-gel method was implied, and various reagents (calcium nitrate tetrahydrate, diammonium hydrogen phosphate, and magnesium nitrate hexahydrate) with higher purity (≥99.0%) were acquired from Sigma Aldrich (St. Louis, MO, USA). The ammonium hydroxide, chitosan, and ethanol were also procured from Sigma Aldrich.

#### Magnesium-Doped Hydroxyapatite in Chitosan Matrix

Magnesium-doped hydroxyapatite suspensions in the chitosan matrix were obtained by the sol-gel technique [40]. The [Ca + Mg]/P ratio was 1.67 and x_Mg_ = 0.05. The solutions of Mg(NO_3_)_2_∙6H_2_O and Ca(NO_3_)_2_∙4H_2_O were dropped into the solutions of (NH_4_)_2_∙HPO_4_ and C_6_H_11_NO_4_. The dripping was made under continuous stirring at 90 °C. During dripping, the pH was kept constant at 10. The suspension obtained after 5 h of stirring after the end of dripping was centrifuged and finally redispersed. This procedure was repeated five times. At the end, the precipitate was redispersed in a 1% chitosan solution and subjected to strong agitation for 12 h. Both the physico-chemical and biological features of the final suspension, called MgHApC, were evaluated.

### 2.2. Characterization Methods

#### 2.2.1. Physico-Chemical Characterization

The stability of the suspension was evaluated by ultrasonic studies [41,42] and the dynamic light scattering (DLS) method. The container of cubic form has two ultrasonic transducers penetrating from the middle of opposite faces. The central frequency of these transducers is 25 MHz. The suspension is stirred for good homogeneity for 5 min. Immediately after, the ultrasonic signals traversing the suspension are acquired, which are recorded every 5 s for a total duration of 5000 s. The recorded amplitudes of the transmitted signals are determined as ratios to the amplitudes in double-distilled water in identical conditions, which are taken as reference liquids. The dynamic light scattering (DLS) method was used to evaluate the average hydrodynamic diameter of magnesium-doped hydroxyapatite suspensions in the chitosan matrix. The nanoparticle analyzer SZ-100 (Horiba-SAS France, Longjumeau, France) was used for DLS measurements. DLS assays were performed in triplicate. The temperature at which the measurements were made was 25 ± 1 °C. The morphology of MgHApC particles was evaluated by scanning electron microscopy. For this purpose, a FEI Quanta Inspect F microscope (FEI Company, Hillsboro, Oregon, United States) was used. The elemental analysis of MgHApC particles was performed by energy dispersive X-ray (EDX) measurements. The high-resolution scanning electron microscopy (HRSEM) micrographs allowed us to achieve particle size distribution. The 3D HRSEM micrographs and 3D images of the elemental mapping cartographies were realized using ImageJ software (Image J 1.51j8) [43].

The Fourier-transform infrared (FTIR) spectra of MgHApC nanoparticles were obtained using a SP 100 Perkin Elmer FTIR spectrometer (Waltham, MS, USA) in agreement with the previous work [40,44]. FTIR studies were carried out to highlight the presence of functional groups characteristic of hydroxyapatite and/or chitosan structures in the analyzed samples.

#### 2.2.2. *In vitro* Antimicrobial Assays

*Staphylococcus aureus* ATCC 25923, *Escherichia coli* ATCC 25922, *Pseudomonas aeruginosa* ATCC 27853, and *Candida albicans* ATCC 10231 microbial strains were used in order to evaluate the antimicrobial activity of MgHApC, MgHAp, HApC, and HAp suspensions. The methodology used for the *in vitro* evaluation of the antimicrobial activity of the samples was described in detail in [40]. The samples were exposed to microbial suspensions (1.5 mL) with a density of approximately 5 × 10^6^ CFU/mL (colony forming units/mL). Phosphate-buffered saline (PBS) was used to prepare the microbial suspensions, which were exposed for different time intervals (24, 48, and 72 h) with the MgHApC, MgHAp, HApC, and HAp suspensions. The CFU/mL values were determined after each time interval. The antimicrobial assays were carried out in triplicate, and the data were depicted graphically as mean ± standard deviation (SD). The statistical analysis was performed using the ANOVA single-factor test.

#### 2.2.3. Cytotoxicity Assay

The cytotoxicity of MgHApC suspensions was investigated using the Human colon cancer HCT-8 cell line. The cells were cultured at a temperature of 37 °C in an atmosphere comprised of 95% O_2_ and 5% CO_2_ in a Complete Medium, enriched with penicillin G and streptomycin. The cell passages were carried out every 5 days. The HCT-8 cells were cultured in 24-well plates, and they were left to adhere and proliferate in the culture medium for 24 h before being exposed to the MgHApC suspensions. After 24 h, they were exposed for 24, 48, and 72 h to the MgHApC suspensions, and the cell viability was determined using the MTT 3-(4,5-dimethyl-thiazolzyl)-2,5-diphenyltetrazolium bromide assay as previously reported by Mosmann [45].

## 3. Results

**Figure 1:**
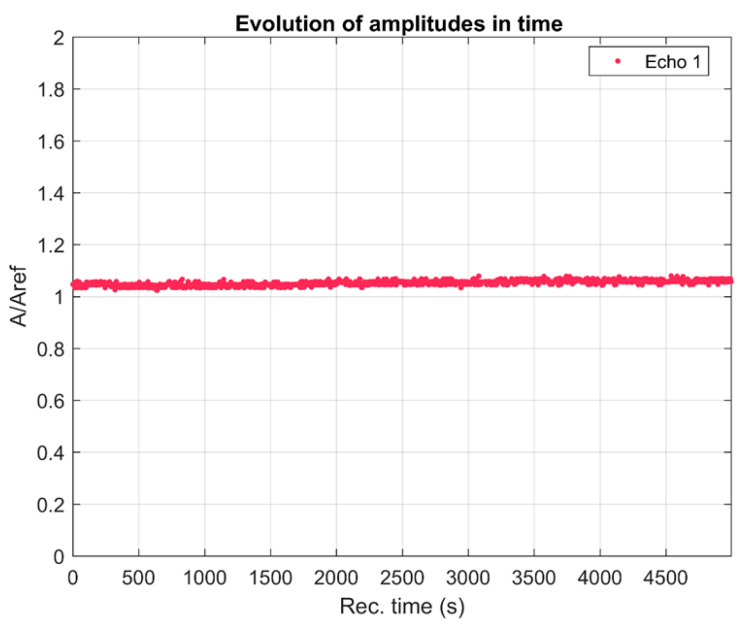
The time evolution of the ultrasonic signal relative amplitudes through the MgHApC suspension.

**Figure 2:**
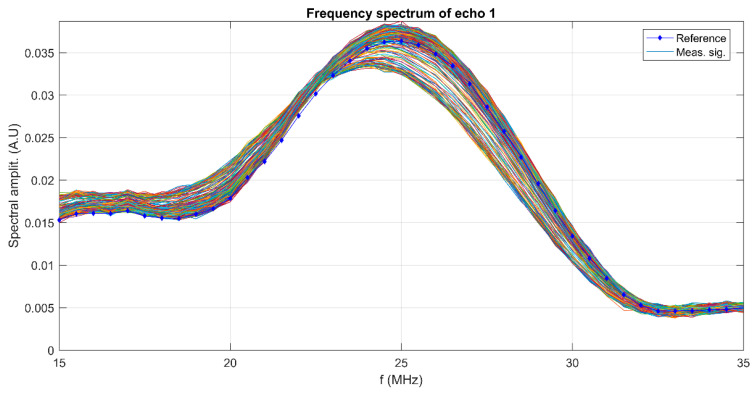
Frequency spectra of the recorded signal for the MgHApC suspension and the reference liquid.

The realization of a biomaterial with potential antimicrobial activity represents an alternative strategy for the prevention of postoperative infections. Implant infections developed by infectious microorganisms remain the most important problem in post-orthopedic surgery. The stability of suspensions, the size of particles on the nanometric scale, and the size of the distribution of particles are very important parameters in the development of innovative materials with high efficiency. The stability of the MgHApC suspensions developed in this study was evaluated by ultrasonic measurements using double-distilled water as the reference, known to be the most stable fluid. An advantage of the method used to evaluate the stability of the developed suspension was that this study could be performed on the concentrated sample (as it was obtained after the synthesis process). In Figure 1, the relative amplitudes of the tested sample are shown. The amplitudes are all higher than in the reference liquid and show a slow, monotonic increase in time. The recorded ultrasonic signals transmitted through the sample are decomposed into a superposition of signals of determined frequency and corresponding amplitudes. These amplitudes are superposed for all the 1000 recorded signals (Figure 2) at the same selected frequencies. The maximum amplitudes are near the central frequency of 25 MHz, which is the central frequency of the transducers, and correspond to the curves obtained for the reference liquid. It can be remarked that for frequencies below 23 MHz, for all recorded moments, the amplitudes of the signals are higher than in the reference liquid. However, above this frequency, there is a stronger scattering of amplitudes in time, showing an important evolution in time from amplitudes that are lower than those in the reference liquid to amplitudes surpassing those in the reference liquid.

**Figure 3:**
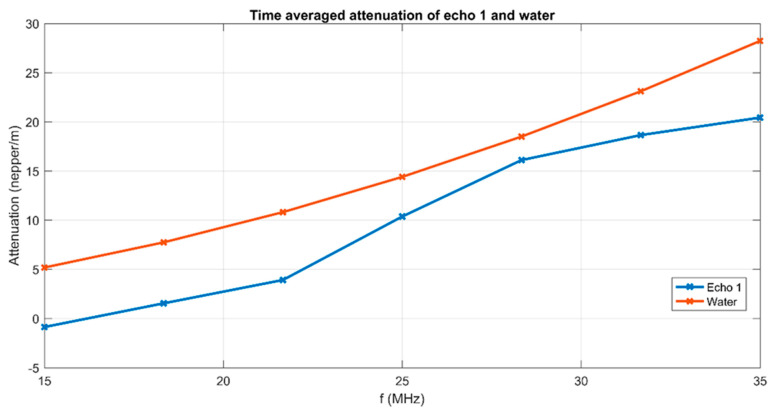
Attenuation of ultrasonic signals vs. frequency for MgHApC suspension.

The attenuation of signals at a range of frequencies centered on 25 MHz is averaged over the 1000 time records (Figure 3). An averaged lower attenuation than in the reference liquid is noted for all frequencies between 15 MHz and 30 MHz. This can be explained by the presence of MgHApC particles in suspension. The averaged stability parameter, $S=\frac{\bar{dA}}{A\;dt}$ suggested by the slope of the interpolation line in Figure 1 is *S* = 6.21991 × 10^−6^ (1/s), which represents very good stability.

**Figure 4:**
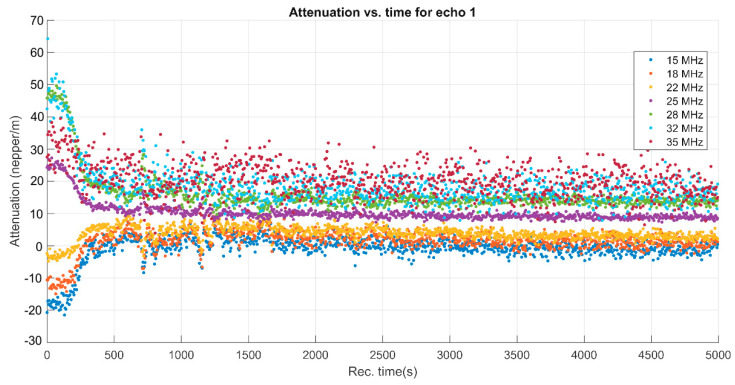
Attenuation of ultrasonic signals vs. time for MgHApC suspension.

The variability of spectral amplitudes shown in Figure 1 indicated the usefulness of showing the evolution of attenuation in the sample for several frequencies during the experiment (Figure 4).

A remarkable phenomenon can be observed during the first 300 s of sedimentation. The attenuation for the lower frequencies (15, 18, 22 MHz) increases rapidly, then slowly decreases. On the contrary, the attenuation determined for the spectral amplitudes at higher frequencies (25, 28, 32, 35 MHz) drops sharply in the same lapse of time, followed by a slow decrease of the attenuation until the end of the experiment. The explanation comes from an initial aggregation of the larger particles, followed by a slow, gradual reduction of the attenuation over time. This information comes as a complement to the ultrasonic signal amplitude (Figure 1), which is not variable during the first 300 s.

Both the size of the particles and the size of their distribution are important parameters for the materials used in the medical field.

**Figure 5:**
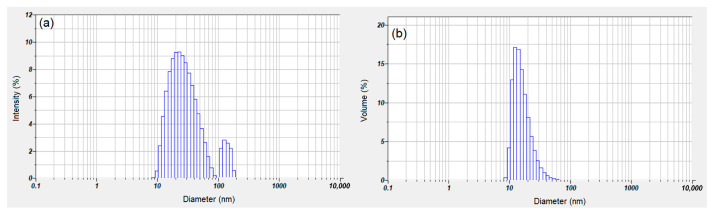
Intensity size distribution (**a**) and volume size distribution (**b**) of suspension MgHApC.

Dynamic light scattering (DLS) studies were conducted for the characterization of suspension MgHApC. Figure 5 shows the intensity particle size distribution achieved for the suspension MgHApC sample. The hydrodynamic diameter and size distribution of MgHApC were obtained. The plot revealed the relative percentage of light scattered by the particle. The two distinct peaks at 29.5 nm and 115 nm indicate a bimodal distribution (Figure 5a). The presence of a distinct peak at 115 nm revealed the presence of aggregates within the samples. When this distribution was converted to a volume-based distribution (Figure 5b), it was evident that the concentration of aggregates was relatively low in the sample. This transformation was performed using the instrument software using the Mie theory of light scattering and the refractive index and absorption of the particles. The volume size distribution indicates that, on a mass basis, the MgHApC suspension consists of small particles of about 18.2 nm.

**Figure 6:**
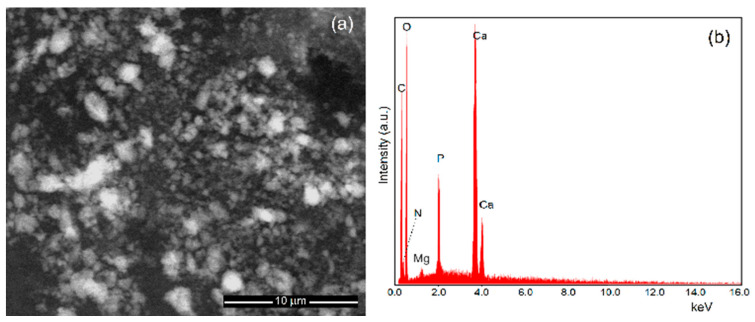
Low magnification SEM micrographs (**a**) and EDS spectrum (**b**) of MgHApC particles.

**Figure 7:**
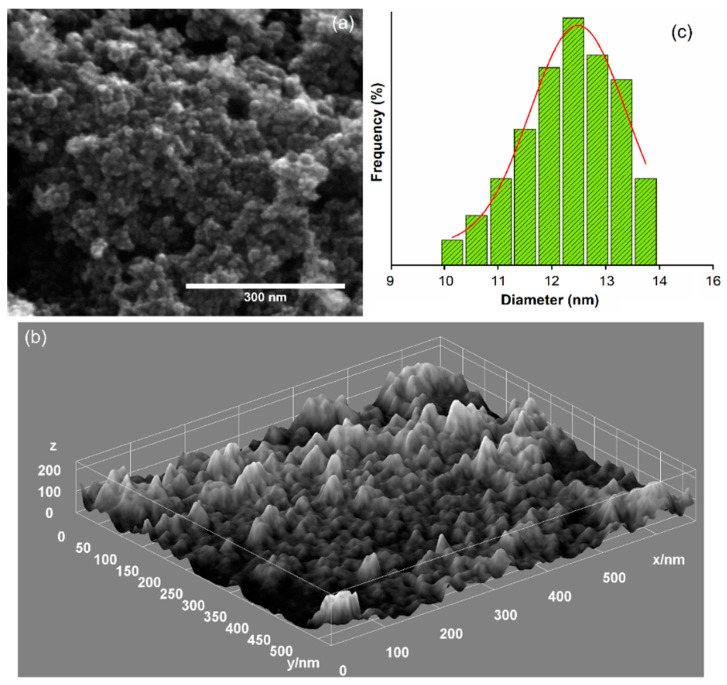
HRSEM micrographs (**a**) and 3D HRSEM micrographs of MgHApC particles (**b**). Size distribution of the MgHApC particles (**c**).

Scanning electron microscopy is the second method used in this study to determine the size of the particles and the size of their distribution. Moreover, SEM studies allowed us to evaluate the morphology of MgHApC particles. Figure 6 shows the micrographs of MgHApC particles recorded at low magnifications and their corresponding EDS spectra. Figure 7 shows the high-resolution scanning electron microscopy (HRSEM) micrographs together with 3D HRSEM micrographs of MgHApC particles. The size distribution of the MgHApC particles is presented in Figure 7c. It can be seen that the MgHApC particles have a spherical shape (Figure 6a and Figure 7a). The spherical shape of MgHApC particles could be due to chitosan. MgHApC particles with a spherical shape present better applicability because there is no risk of puncturing the cell membrane when they are used in various medical applications, such as implants. The average size of the MgHApC particles determined after counting more than 700 particles was 12.5 ± 2 nm (Figure 7c). The EDS spectrum of the MgHApC particles presented in Figure 6b highlights the presence of the elements phosphorus, calcium, magnesium, oxygen, nitrogen, and carbon. It is noteworthy that no additional lines specific to other elements were detected in the EDS spectrum of MgHApC.

**Figure 8:**
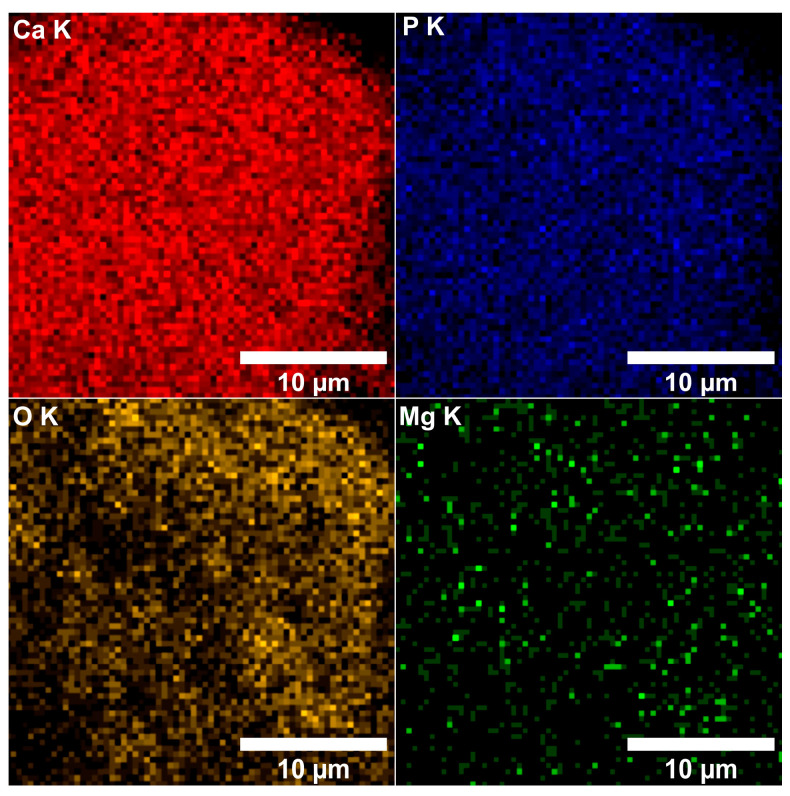
Elemental mapping cartographies were obtained for the MgHApC sample.

**Figure 9:**
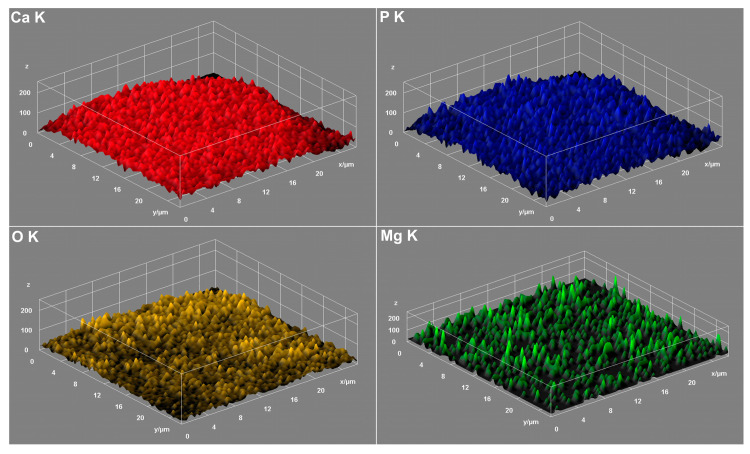
Three-dimensional representation of elemental mapping cartographies obtained for MgHApC.

By performing elemental EDS mapping studies, we analyzed if there was a homogeneous distribution of magnesium within the MgHApC sample. Therefore, in Figure 8 and Figure 9, both 2D and 3D representations of the elemental mapping cartographies obtained for the main constituents’ elements that could be found in the MgHApC sample were revealed. The results of elemental EDS mapping show that in the synthesized MgHApC sample, calcium, oxygen, phosphorus, and magnesium are homogeneously and uniformly distributed. The 3D images (Figure 9) sustain and confirm these results.

**Figure 10:**
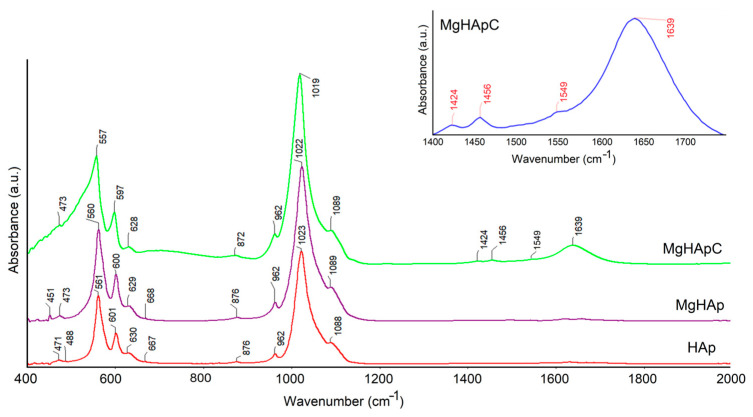
Typical absorbance FTIR spectra of MgHApC, MgHAp, and HAp samples.

Figure 10 shows the absorbance FTIR spectra of MgHApC, magnesium-doped hydroxyapatite (MgHAp), and hydroxyapatite (HAp) samples. In what follows, we will only discuss the results obtained for the MgHApC sample. The band found at around 628 cm^−1^ is typical for structural hydroxyl groups (OH^−^) from the HAp [46,47,48,49]. The –CH_2_^–^ bending vibration (from the chitosan structure) was found at 1424 cm^−1^ [46,47,48,49]. Furthermore, the 1639 and 1456 cm^−1^ maxima are indicating the presence of amide I (C=O) and amino (NH_2_) groups from the chitosan structure in the MgHApC sample [46,47,48,49,50]. According to the studies conducted by Chatterjee N.S. and collaborators, the maxima found at 1549 cm^−1^ suggest the presence of amide II groups from the chitosan structure in the MgHApC sample [51]. The absorption bands observed around 1089 (ν_3_), 1019 (ν_3_), 962 (ν_1_), 597 (ν_4_), 557 (ν_4_), and 473 cm^−1^ (ν_2_) can be attributed to the phosphate group from the apatite structure present in the MgHApC sample [47]. The presence of the maxima located at 872 may suggest the presence of HPO_4_^2−^ or CO_3_^2−^ in the studied sample [40,41,44,46]. Moreover, in the FTIR spectra obtained for the MgHAp and HAp samples, only the presence of the maxima that belong to the functional groups (phosphate and hydroxyl) from the hydroxyapatite can be noticed. The presence of chitosan in the samples induces a broadening of the maxima along with their slight displacement towards lower values. This behavior is in agreement with previous studies [47,49]. The shift towards lower values of the maxima demonstrates the interaction of chitosan with hydroxyapatite. In the inset from Figure 10, it is presented in detail the spectral region where the maxima are specific to the chitosan [46,47,48,49].

**Figure 11:**
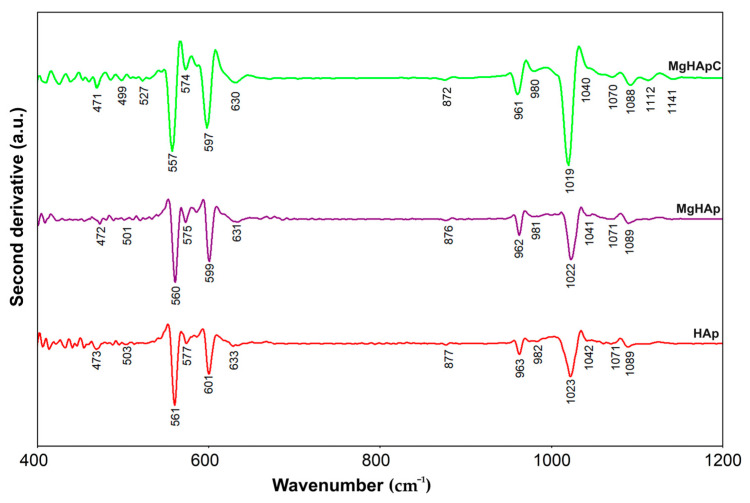
FTIR—second derivative spectra obtained for the MgHApC, MgHAp, and HAp samples.

Furthermore, additional information was obtained by performing FTIR second derivative studies. Figure 11 reveals the FTIR second derivative spectra characteristic for the MgHApC, MgHAp, and HAp samples in the 400–1200 cm^−1^ spectral domain.

In the case of the MgHApC sample, all the maxima that are found in Figure 11 mainly belong to the “fingerprint” vibration domain of (PO_4_^3−^) groups from hydroxyapatite. The absorption bands noticed at 471 and 499 cm^−1^ are specific to the ν_2_ vibration of the phosphate group in apatite [52]. Furthermore, the intense adsorption bands observed at 527, 557, 574, and around 597 cm^−1^ are in the ν_4_ vibration domain of the phosphate group. The most intense phosphate band that could be noticed in Figure 11 is located at 1019 cm^−1^ in the ν_3_ (PO_4_^3−^) domain. Also, the adsorption bands specific to the ν_3_ and ν_1_ domains of phosphate groups from the hydroxyapatite structure are found at around 961–980 cm^−1^ (ν_1_) and in the 1019–1200 cm^−1^ (ν_3_) spectral domain [53]. According to the previous studies, the narrow shoulder found at 872 cm^−1^ could be attributed to the CO_3_^2−^ group (B type) [53] or to the presence of HPO_4_^2−^ in the MgHApC sample [46,47,48,49]. For MgHApC, the librational mode of hydroxyl groups at 630 cm^−1^ is clearly visible in the FTIR-second derivative spectra. As can be seen in Figure 11, similar results were obtained for MgHAp and HAp samples. As can be seen, the presence of chitosan induces a broadening of the maxima and their displacement towards lower values [47,49].

**Figure 12:**
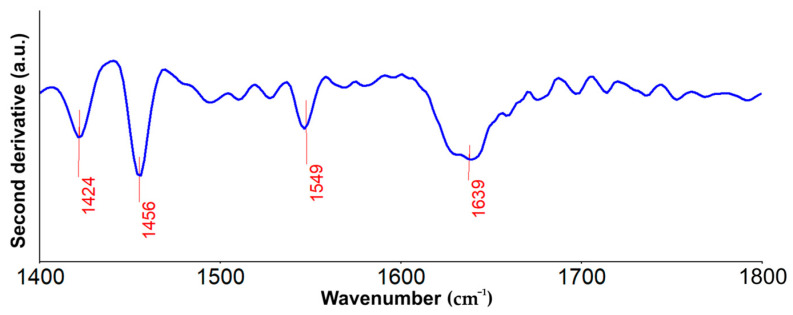
FTIR—second derivative spectra obtained for the MgHApC sample in the 1400–1800 cm^−1^ spectral domain.

Furthermore, in Figure 12, the second derivative spectra obtained for the MgHApC sample are presented in the 1400–1800 cm^−1^ spectral domain. Herein could be noticed the presence of the bands that may be associated with the –CH_2_– bending vibration (around 1424 cm^−1^), amino (around 1456 cm^−1^), amide II (around 1549 cm^−1^), and amide I (around 1639 cm^−1^), groups from chitosan [46,47,48,49,50].

More than that, no other important adsorption bands could be identified in the studied spectral domains, which may suggest the purity of the analyzed sample. These results are in concordance with the data reported by Kolmas J. et al. [54] and Nawrotek K. and collaborators [53] concerning the FTIR characteristics of the hydroxyapatite biocomposites. Therefore, the results of the second derivative-FTIR analysis sustain and confirm the findings of the FTIR studies performed on the MgHApC, MgHAp, and HAp samples.

**Figure 13:**
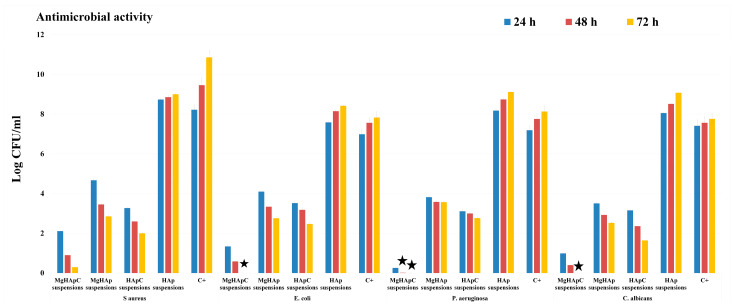
The graphical representation of the antimicrobial activity of MgHApC, MgHAp, HApC, and HAp suspensions against *Staphylococcus aureus* ATCC 25923, *Escherichia coli* ATCC 25922, *Pseudomonas aeruginosa* ATCC 27853, and *Candida albicans* ATCC 10231 microbial strains after 24, 48, and 72 h of incubation.

Nowadays, the advances made in the scientific field of biomedical applications have allowed the development of novel biomaterials that are both effective and safe to use. Nonetheless, there are significant imitations in the current biomaterials used in various applications, most notably in dentistry. In the dentistry field, the major issues regarding the materials used are represented by the acute inflammatory responses triggered by the presence of dental restorative materials. It has been reported that if the formation of the dentinal bridge takes place inside the teeth, that will lead to irreversible inflammation, which will require a complex plan of treatment at the cost of both the patient and the health care provider [7,55,56]. In this context, the aim of this work was to develop a biomaterial with improved restorative properties and antimicrobial activity that could be used successfully for biomedical applications. Furthermore, quantitative assays regarding the antimicrobial activity of the MgHApC, MgHAp, HApC, and HAp suspensions were performed. The antimicrobial activity of the MgHApC, MgHAp, HApC, and HAp suspensions was studied against one of the most common microbial strains that are often responsible for patient infections: *S. aureus*, *E. coli*, *P. aeruginosa*, and *C. albicans*. For this purpose, the MgHApC suspensions as well as the MgHAp, HApC, and HAp suspensions were incubated with the microbial suspensions, and the number of CFU/mL was quantified after 24, 48, and 72 h of incubation. The experiments were conducted in triplicate, and the results were depicted graphically as mean ± SD in Figure 13.

The results of the *in vitro* antimicrobial assays demonstrated MgHApC suspensions exhibited a strong bacteriostatic effect even after 24 h of incubation. Furthermore, the data revealed that the suspensions exhibited bactericidal properties after 48 h of incubation for some of the tested microbial strains. The results highlighted that the antimicrobial activity of the MgHApC suspensions was correlated with the exposure time. Therefore, after 72 h of exposure, the MgHApC suspensions demonstrated both bactericidal and fungicidal activity against *E. coli*, *P. aeruginosa*, and *C. albicans*. In addition, the results also highlighted that the most susceptible microbial strain to the MgHApC suspensions was *P. aeruginosa* for all tested time intervals. In addition, the antimicrobial activity of HAp suspensions as well as MgHAp and HApC suspensions was evaluated. The results of the *in vitro* antimicrobial assays demonstrated that HAp suspensions did not negatively affect the development of any of the tested microbial strains. On the contrary, the exposure to the HAp suspensions was responsible for an augmentation in the development of all the tested microbial strains compared to the control culture (C+). The results suggested that the increase in the CFU number in the case of HAp suspensions compared to the control was also influenced by the exposure time. The data also highlighted that the HAp suspensions promoted the increase of the CFU number differently depending on the microbial strain. Nonetheless, regardless of the microbial strain, the increase in the CFU number was time-dependent. Furthermore, the results regarding the antimicrobial activity exhibited by the MgHAp suspensions are also depicted in Figure 13. The results emphasized that even at low concentrations of magnesium, the MgHAp suspensions exhibited good bacteriostatic properties for all the tested microbial strains. In addition, HApC suspensions were also investigated for their antimicrobial activity. The results revealed that they presented better antimicrobial activity than the MgHAp suspensions compared to the control (C+) but that they exhibited a lower inhibition rate than the MgHApC suspensions. More than that, the results also indicated that the antimicrobial activity was correlated with the incubation time. This behavior suggests that the antimicrobial activity of the MgHAp suspensions is attributed to the presence of magnesium ions and to the synergy that is created between the magnesium ions and the hydroxyapatite matrix. More than these results, they also highlight the premises of a slow release of magnesium ions from the hydroxyapatite matrix. The results obtained are in good agreement with previously reported data regarding the antimicrobial activity of biocomposites based on hydroxyapatite with magnesium and chitosan [40,56,57,58,59,60,61,62]. Furthermore, the data also suggest that the enhanced antimicrobial activity (bactericidal and fungicidal) exhibited by the MgHApC suspensions could be attributed to the presence of magnesium ions and chitosan, as well as the synergies that appear between them and the hydroxyapatite matrix. These results are in good concordance with previous reported studies regarding the antimicrobial properties of chitosan and chitosan/hydroxyapatite compounds [40,62,63,64,65]. Even though there is information regarding the antimicrobial mechanism of novel biocomposites, the effects that they have against microorganisms are attributed to both their individual physico-chemical and biological properties as well as to the synergies that appear between the components of the biocomposites [40,54,55,56,57,58,59,60,61,62,63,64,65,66]. In this case, the enhanced biological properties of HAp are well known and documented, and the use of chitosan in dentistry applications has been the subject of research for some years. Due to its unique features and the possibility of creating a gel or hydrogel, chitosan could be used in the treatment of chronic periodontitis as well as canker sores. Chitosan was reported to possess antibacterial activity against some of the most common bacterial strains deemed responsible for dental plaque formation: *Streptococcus mutans*, *Actinomyces actinomycetemcomitans*, and *Porphyromonas gingivalis* [67,68]. Moreover, some studies have shown that chitosan-based toothpastes, mouthwashes, and even chewing gum could lead to a significant reduction of *S. mutans* bacterial cells in the oral cavity [69,70]. Currently, chitosan is being investigated for its antimicrobial activity in various applications in food packaging, fabrics, and cosmetic industries, as well as in biomedical applications, including dentistry [71,72,73]. It has been reported that the mechanism through which chitosan manages to inhibit bacteria is mostly based on bacteriostatic routes and that its antimicrobial properties are correlated to its degree of de-acetylation and also to the molecular weight [72,73]. On the other hand, according to Kong et al. [68], there are four main categories of parameters that could have an influence on the antimicrobial activity of chitosan: the microorganisms (type of microbial cells, its stage of life, etc.); some intrinsic properties of chitosan (charge density, molecular weight, hydrophobic and hydrophilic characteristics, chelation capacity); the physical state factors in which chitosan is used (soluble or solid state); and the environmental parameters (pH, ionic forces, temperature, time). More than that, both magnesium-doped hydroxyapatite and chitosan are materials that have excellent biocompatibility and bioactivity, thus making them suitable candidates for the development of novel biocomposites for future applications in medicine, dentistry, food packaging, prosthesis, etc.

**Figure 14:**
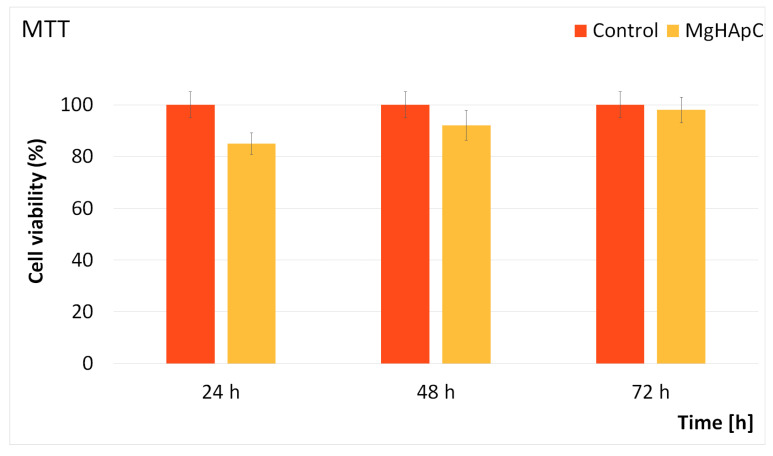
MTT assay of HCT-8 cells incubated with MgHApC suspensions for 24, 48, and 72 h. *p* ≤ 0.05 was accepted as statistically significant.

Supplementary information about the biological properties of the MgHApC suspensions was gathered by evaluating the cytotoxicity of MgHApC with the aid of HCT-8 cells. For this purpose, HCT-8 cells were exposed to MgHApC suspensions for three different time intervals, and their cell viability was evaluated using a MTT test. The results of the MTT assay depicted in Figure 14 revealed that the cell viability of the HCT-8 cells remained above 85% after being exposed to the MgHApC suspensions for 24 h. Moreover, the data also emphasized that after 48 h and 72 h of exposure, the cell viability of the HCT-8 cells increased, reaching 92% and 98%, respectively. These results are in good agreement with other studies on the topic of the biological properties of biocomposites based on magnesium-doped hydroxyapatite [40,52,74,75,76].

Magnesium ions are considered to be of great importance in the most important regulatory processes in the human organism. In their study, Bigi et al. [77] showed that magnesium ions are responsible for the formation of new bone mineral nuclei. In addition, Boanini et al. [78] established that magnesium ions have the role of regulators in osteoblast cells, having a good effect in the case of osteoporotic bones. Therefore, the results obtained in our study regarding the synthesis, physico-chemical, and biological evaluation of a novel material based on magnesium-doped hydroxyapatite in a chitosan matrix will contribute greatly to the progress of the innovative materials domain. The preliminary results obtained in this study offer great opportunities for the future development of novel magnesium doped hydroxyapatite in chitosan matrix-based products.

The present research revealed concrete results that can lead to the development of new implantable materials such as prostheses, screws (in orthopedics), and pivots (in dentistry) covered with MgHApC. Coated implantable devices can have a positive impact on patients by decreasing post-operative or post-interventional infections as a result of their antimicrobial and non-cytotoxic characteristics. Future research will aim to study the physico-chemical and biological properties of mixtures based on MgHApC and materials used in dental fillings.

## 4. Conclusions

In the present work, a nontoxic magnesium-doped hydroxyapatite in chitosan (MgHApC) matrix was developed efficaciously in the laboratory by an adapted co-precipitation method and characterized. The FTIR measurement results highlighted the presence of both typical vibration bands of the HAp and those of the chitosan structure. The MgHApC nanoparticles obtained in the laboratory exhibited excellent antimicrobial activity against all the tested microorganisms (*Escherichia coli* ATCC 25922, *Staphylococcus aureus* ATCC 25923, *Pseudomonas aeruginosa* ATCC 27853, and *Candida albicans* ATCC 10231). More than that, the results of the *in vitro* assays demonstrated that the antimicrobial properties of MgHApC increased with an increase in exposure time for all the tested microorganisms. In addition, the results of the *in vitro* antimicrobial experiments also showed that the most susceptible microbial strain to MgHApC nanoparticles was *Pseudomonas aeruginosa* ATCC 27853. The results of the cytotoxicity evaluation (using the Human colon cancer (HCT-8) cell line) showed that MgHApC nanoparticles exhibit a nontoxic effect. The present study showed that non-toxic MgHApC nanoparticles obtained in the laboratory at low cost could be very useful and applicable in the future in several fields, such as the food industry (as a preservative agent), medicine (in implant coatings to decrease the risk of postoperative infections), and dentistry. Based on the obtained results, this study gives us the chance to carry out further complex research in the future.

## Data Availability

Data available on demand from the corresponding author.

## References

[1] Fakhri E., Eslami H., Maroufi P., Pakdel F., Taghizadeh S., Ganbarov K., Yousefi M., Tanomandf A., Yousefi B., Mahmoudi S. (2020). Chitosan biomaterials application in dentistry. Int. J. Biol. Macromol..

[2] Mehrabani M.G., Karimian R., Mehramouz B., Rahimi M., Kafil H.S. (2018). Preparation of biocompatible and biodegradable silk fibroin/chitin/silver nanoparticles 3D scaffolds as a bandage for antimicrobial wound dressing. Int. J. Biol. Macromol..

[3] Chan B., Leong K. (2008). Scaffolding in tissue engineering: General approaches and tissue specific considerations. Eur. Spine J..

[4] Cross L.M., Thakur A., Jalili N.A., Detamore M., Gaharwar A.K. (2016). Nanoengineered biomaterials for repair and regeneration of orthopedic tissue interfaces. Acta Biomater..

[5] Liang J., Peng X., Zhou X., Zou J., Cheng L. (2020). Emerging Applications of Drug Delivery Systems in Oral Infectious Diseases Prevention and Treatment. Molecules.

[6] Qin Y., Li P. (2020). Antimicrobial chitosan conjugates: Current synthetic strategies and potential applications. Int. J. Mol. Sci..

[7] Salem R.M., Zhang C., Chou L. (2021). Effect of Magnesium on Dentinogenesis of Human Dental Pulp Cells. Int. J. Biomater..

[8] Sharifianjazi F., Khaksar S., Esmaeilkhanian A., Bazli L., Eskandarinezhad S., Salahshour P., Sadeghi F., Rostamnia S., Vahdat S.M. (2022). Advancements in Fabrication and Application of Chitosan Composites in Implants and Dentistry: A Review. Biomolecules.

[9] Gupta A., Pratt R., Mishra B. (2018). Physicochemical characterization of ferric pyrophosphate citrate. Biometals.

[10] Subramaniyan S., Kamaraj Y., Kumaresan V., Kannaiyan M., David E., Ranganathan B., Selvaraj V., Balupillai A. (2022). Green synthesized zinc oxide nanoparticles induce apoptosis by suppressing PI3K/Akt/mTOR signaling pathway in osteosarcoma MG63 cells. Glob. Transl. Med..

[11] Karahan M., Karahan N., Ozkan F., Yildirim K. (2021). Characterization of Natural Reinforcements and their Composites. J. Compos. Biodegrad. Polym..

[12] Gosain A.K., Plastic Surgery Eductional Foundation DATA Committee (2005). Biomaterials for reconstruction of the cranial vault. Plast. Reconstr. Surg..

[13] Ozawa T., Mickle D.A., Weisel R.D., Koyama N., Ozawa S., Li R.-K. (2002). Optimal biomaterial for creation of autologous cardiac grafts. Circulation.

[14] Zafar M.S., Alnazzawi A.A., Alrahabi M., Fareed M.A., Najeeb S., Khurshid Z. (2019). Nanotechnology and nanomaterials in dentistry. Advanced Dental Biomaterials.

[15] Vallet-Regi M., González-Calbet J.M. (2004). Calcium phosphates as substitution of bone tissues. Prog. Solid State Chem..

[16] Siddiqui N., Madala S., Parcha S.R., Mallick S.P. (2020). Osteogenic differentiation ability of human mesenchymal stem cells on Chitosan/Poly(Caprolactone)/nano beta Tricalcium Phosphate composite scaffolds. Biomed. Phys. Eng. Express.

[17] Joshy M.A., Kolanthai E., Kumar V.S., Sindu P.A., Asokan K., Kalkura S.N. (2021). Investigations on the effect of swift heavy silicon ion irradiation on hydroxyapatite. Mater. Today Proc..

[18] Abutalib M.M., Yahia I.S. (2017). Novel and facile microwave-assisted synthesis of Mo-doped hydroxyapatite nanorods: Characterization, gamma absorption coefficient, and bioactivity. Mater. Sci. Eng. C.

[19] Li Z., Chu D., Gao Y., Jin L., Zhang X., Cui W., Li J. (2019). Biomimicry, biomineralization, and bioregeneration of bone using advanced three-dimensional fi-brous hydroxyapatite scaffold. Mater. Today Adv..

[20] Nesseri E., Boyatzis S.C., Boukos N., Panagiaris G. (2020). Optimizing the biomimetic synthesis of hydroxyapatite for the consolidation of bone using diammonium phosphate, simulated body fluid, and gelatin. SN Appl. Sci..

[21] Morris H.F., Ochi S. (1998). Hydroxyapatite-coated implants: A case for their use. J. Oral Maxillofac. Surg..

[22] Luo J., Mamat B., Yue Z., Zhang N., Xu X., Li Y., Su Z., Ma C., Zang F., Wang Y. (2021). Multi-metal ions doped hydroxyapatite coatings via electrochemical methods for antibacterial and osteogenesis. Colloids Interface Sci. Commun..

[23] Predoi D., Ciobanu S.C., Iconaru S.L., Predoi M.V. (2023). Influence of the Biological Medium on the Properties of Magnesium Doped Hydroxyapatite Composite Coatings. Coatings.

[24] Luque-Agudo V., Fernández-Calderón M.C., Pacha-Olivenza M.A., Perez-Giraldo C., Gallardo-Moreno A.M., González-Martín M.L. (2020). The role of magnesium in biomaterials related infections. Colloids Surf. B.

[25] Kis V.K., Sulyok A., Hegedus M., Kovács I., Rózsa N., Kovács Z. (2021). Magnesium incorporation into primary dental enamel and its effect on mechanical properties. Acta Biomater..

[26] Ding H., Pan H., Xu X., Tang R. (2014). Toward a detailed understanding of magnesium ions on hydroxyapatite crystallization inhibition. Cryst. Growth Des..

[27] Sionkowska A. (2011). Current research on the blends of natural and synthetic polymers as new biomaterials. Prog. Polym. Sci..

[28] Song R., Murphy M., Li C., Ting K., Soo C., Zheng Z. (2018). Current development of biodegradable polymeric materials for biomedical applications. Drug Des. Dev. Ther..

[29] Prashanth K.H., Tharanathan R. (2007). Chitin/chitosan: Modifications and their unlimited application potential—An overview. Trends Food Sci. Technol..

[30] Rahimi M., Ahmadi R., Kafil H.S., Shafiei-Irannejad V. (2019). A novel bioactive quaternized chitosan and its silver containing nanocomposites as a potent antimicrobial wound dressing: Structural and biological properties. Mater. Sci. Eng..

[31] Tshinyangu K.K., Hennebert G.L. (1996). Protein and chitin nitrogen contents and protein content in Pleurotus ostreatus var. columbinus. Food Chem..

[32] Pillai C., Paul W., Sharma C.P. (2009). Chitin and chitosan polymers: Chemistry, solubility and fiber formation. Prog. Polym. Sci..

[33] Bakshia P.S., Selvakumara D., Kadirvelub K., Kumara N. (2019). Chitosan as an environment friendly biomaterial–a review on recent modifications and applications. Int. J. Biol. Macromol..

[34] Zhao S., Jiang Q., Peel S., Wang X., He F. (2013). Effects of magnesium-substituted nanohydroxyapatite coating on implant osseointegration. Clin. Oral Implant. Res..

[35] Harrel S.K., Molinari J. (2004). Aerosols and splatter in dentistry: A brief review of the literature and infection control implications. J. Am. Dent. Assoc..

[36] Mulazzi M., Campodoni E., Bassi G., Montesi M., Panseri S., Bonvicini F., Gentilomi G.A., Tampieri A., Sandri M. (2021). Medicated Hydroxyapatite/Collagen Hybrid Scaffolds for Bone Regeneration and Local Antimicrobial Therapy to Prevent Bone Infections. Pharmaceutics.

[37] Hu Y., Wan L., Xiao Y., Wang Y., Wu Z., Guo W., Yang H., Hu T. (2022). Enhanced reparative dentinogenesis of biphasic calcium phosphate ceramics containing calcium-deficient hydroxyapatite (CDHA) and strontium-incorporated CDHA in direct pulp capping. Mater. Today Commun..

[38] Li F.-Y., Chaigne-Delalande B., Kanellopoulou C. (2011). Second messenger role for Mg^2+^ revealed by human T cell immunodeficiency. Nature.

[39] Jenifer A., Senthilarasan K., Arumugam S., Sivaprakash P., Sagadevan S., Sakthivel P. (2021). Investigation on antibacterial and hemolytic properties of magnesium-doped hydroxyapatite nanocomposite. Chem. Phys. Lett..

[40] Predoi D., Ciobanu C.S., Iconaru S.L., Predoi S.A., Chifiriuc M.C., Raaen S., Badea M.L., Rokosz K. (2022). Impact of Gamma Irradiation on the Properties of Magnesium-Doped Hydroxyapatite in Chitosan Matrix. Materials.

[41] Predoi D., Iconaru S.L., Predoi M.V., Motelica-Heino M., Buton N., Megier C. (2020). Obtaining and Characterizing Thin Layers of Magnesium Doped Hydroxyapatite by Dip Coating Procedure. Coatings.

[42] Predoi D., Iconaru S.L., Predoi M.V., Motelica-Heino M., Guegan R., Buton N. (2019). Evaluation of Antibacterial Activity of Zinc-Doped Hydroxyapatite Colloids and Dispersion Stability Using Ultrasounds. Nanomaterials.

[43] ImageJ. http://imagej.nih.gov/ij.

[44] Iconaru S.L., Prodan A.M., Turculet C.S., Beuran M., Ghita R.V., Costescu A., Groza A., Chifiriuc M.C., Chapon P., Gaiaschi S. (2016). Enamel Based Composite Layers Deposited on Titanium Substrate with Antifungal Activity. J. Spectrosc..

[45] Mosmann T. (1983). Rapid colorimetric assay for cellular growth and survival: Application to proliferation and cytotoxicity assays. J. Immun. Met..

[46] Chen J., Nan K., Yin S., Wang Y., Wu T., Zhang Q. (2010). Characterization and biocompatibility of nanohybrid scaffold prepared via in situ crystallization of hydroxyapatite in chitosan matrix. Colloids Surf. B..

[47] Pramanik N., Mishra D., Banerjee I., Maiti T.K., Bhargava P., Pramanik P. (2009). Chemical synthesis, characterization, and biocompatibility study of hydroxyapatite/chitosan phosphate nanocomposite for bone tissue engineering applications. Int. J. Biomater..

[48] Banerjee S., Bagchi B., Bhandary S., Kool A., Hoque N.A., Biswas P., Pal K., Thakur P., Das K., Karmakar P. (2018). Antimicrobial and biocompatible fluorescent hydroxyapatite-chitosan nanocomposite films for biomedical applications. Colloids Surf. B.

[49] Nikpour M.R., Rabiee S.M., Jahanshahi M.J.C.P.B.E. (2012). Synthesis and characterization of hydroxyapatite/chitosan nanocomposite materials for medical engineering applications. Compos. B Eng..

[50] Alanis-Gómez R.P., Rivera-Muñoz E.M., Luna-Barcenas G., Alanis-Gómez J.R., Velázquez-Castillo R. (2022). Improving the Mechanical Resistance of Hydroxyapatite/Chitosan Composite Materials Made of Nanofibers with Crystalline Preferential Orientation. Materials.

[51] Chatterjee N.S., Anandan R., Navitha M., Asha K.K., Kumar K.A., Mathew S., Ravishankar C.N. (2016). Development of thiamine and pyridoxine loaded ferulic acid-grafted chitosan microspheres for dietary supplementation. J. Food Sci. Technol..

[52] Popa C., Ciobanu C., Iconaru S., Stan M., Dinischiotu A., Negrila C., Motelica-Heino M., Guegan R., Predoi D. (2014). Systematic investigation and in vitro biocompatibility studies on mesoporous europium doped hydroxyapatite. Open Chem..

[53] Nawrotek K., Grams J. (2021). Understanding Electrodeposition of Chitosan–Hydroxyapatite Structures for Regeneration of Tubular-Shaped Tissues and Organs. Materials.

[54] Kolmas J., Jaklewicz A., Zima A., Bućko M., Paszkiewicz Z., Lis J., Ślósarczyk A., Kolodziejski W. (2011). Incorporation of carbonate and magnesium ions into synthetic hydroxyapatite: The effect on physicochemical properties. J. Mol. Struct..

[55] Bergenholtz G., Spangberg L. (2004). Controversies in endodontics. Crit. Rev. Oral Biol. Med..

[56] Farges J.-C., Alliot-Licht B., Renard E. (2015). Dental pulp defence and repair mechanisms in dental caries. Mediat. Inflamm..

[57] Iconaru S.L., Ciobanu C.S., Predoi G., Rokosz K., Chifiriuc M.C., Bleotu C., Stanciu G., Hristu R., Raaen S., Raita S.M. (2022). Biological and Physico-Chemical Properties of Composite Layers Based on Magnesium-Doped Hydroxyapatite in Chitosan Matrix. Micromachines.

[58] Ghosh R., Das S., Mallick S.P., Beyene Z. (2022). A Review on the Antimicrobial and Antibiofilm Activity of Doped Hydroxyapatite and its Composites for Biomedical Applications. Mater. Today Commun..

[59] Rabea E.I., Badawy M.E., Stevens C.V., Smagghe G., Steurbaut W. (2003). Chitosan as Antimicrobial Agent: Applications and Mode of Action. Biomacromolecules.

[60] Confederat L.G., Tuchilus C.G., Dragan M., Sha’at M., Dragostin O.M. (2021). Preparation and Antimicrobial Activity of Chitosan and Its Derivatives: A Concise Review. Molecules.

[61] Hans S., Fatima Z., Ahmad A., Hameed S. (2022). Magnesium impairs Candida albicans immune evasion by reduced hyphal damage, enhanced β-glucan exposure and altered vacuole homeostasis. PLoS ONE.

[62] Cicciù M., Fiorillo L., Cervino G. (2019). Chitosan Use in Dentistry: A Systematic Review of Recent Clinical Studies. Mar. Drugs.

[63] Li B., Xia X., Guo M., Jiang Y., Li Y., Zhang Z., Liu S., Li H., Liang C., Wang H. (2019). Biological and antibacterial properties of the micronanostructured hydroxyapatite/chitosan coating on titanium. Sci. Rep..

[64] Morsy R., Ali S.S., El-Shetehy M. (2017). Development of hydroxyapatite-chitosan gel sunscreen combating clinical multidrug-resistant bacteria. J. Mol. Struct..

[65] Costa-Pinto A.R., Lemos A.L., Tavaria F.K., Pintado M. (2021). Chitosan and hydroxyapatite based biomaterials to circumvent periprosthetic joint infections. Materials.

[66] Predoi S.A., Ciobanu S.C., Chifiriuc M.C., Motelica-Heino M., Predoi D., Iconaru S.L. (2023). Hydroxyapatite Nanopowders for Effective Removal of Strontium Ions from Aqueous Solutions. Materials.

[67] Raafat D., Sahl H.-G. (2009). Chitosan and its antimicrobial potential—A critical literature survey. Microb. Biotechnol..

[68] Palma P.J., Ramos J.C., Martins J.B., Diogenes A., Figueiredo M.H., Ferreira P., Viegas C., Santos J.M. (2017). Histologic evaluation of regenerative endodontic procedures with the use of chitosan scaffolds in immature dog teeth with apical periodontitis. J. Endod..

[69] Singla A.K., Chawla M. (2001). Chitosan: Some pharmaceutical and biological aspects--an update. J. Pharm. Pharmacol..

[70] Chavez de Paz L.E., Resin A., Howard K.A., Sutherland D.S., Wejse P.L. (2011). Antimicrobial effect of chitosan nanoparticles on Streptococcus mutans biofilms. Appl. Environ. Microbiol..

[71] ElShiha H.Y., Abdel Monem Tawfik H., Abou Samrah N.K., El Magid Marzouk H.A. (2012). Efficacy of chitosan and absorbable gelatine sponge on hemostasis and wound healing following tooth extraction “A Comparative Study”. Egypt. Dent. J..

[72] Senel S., McClure S.J. (2004). Potential applications of chitosan in veterinary medicine. Adv. Drug Deliv.Rev..

[73] Kenawy E.R., Worley S.D., Broughton R. (2007). The chemistry and application of antimicrobial polymers: A state-of the-art-review. Biomacromolecules.

[74] Predoi D., Ciobanu C.S., Iconaru S.L., Raaen S., Badea M.L., Rokosz K. (2022). Physicochemical and Biological Evaluation of Chitosan-Coated Magnesium-Doped Hydroxyapatite Composite Layers Obtained by Vacuum Deposition. Coatings.

[75] Zhang J., Dai C., Wei J., Wen Z., Zhang S., Lin L. (2013). Calcium phos-phate/chitosan composite coating: Effect of different concentrations of Mg^2+^ in the m-SBF on its bioactivity. Appl. Surf. Sci..

[76] Landi E., Logroscino G., Proietti L., Tampieri A., Sandri M., Sprio S. (2008). Biomimetic Mg-substituted hydroxyapatite: From synthesis to in vivo behaviour. J. Mater. Sci. Mater. Med..

[77] Bigi A., Foresti E., Gregorini R., Ripamonti A., Roveri N., Shah J.S. (1992). The role of magnesium on the structure of biological apatites. Calcif. Tissue Int..

[78] Boanini E., Gazzano M., Bigi A. (2010). Ionic substitutions in calcium phosphates synthesized at low temperature. Acta Biomater..

